# Supplementary material for: A Likelihood Approach for Real-Time Calibration of Stochastic Compartmental Epidemic Models
Source: PLoS Comput Biol. 2017 Jan 17;13(1):e1005257. doi: 10.1371/journal.pcbi.1005257 (PMC5240920; doi:10.1371/journal.pcbi.1005257)
Supplement: S1 File — (TAR.GZ) [file pcbi.1005257.s014.tar.gz › HSPH_Online-SI-Revision/IPoi09/n10-extreme/n10-extreme_BENCH2_table0.pdf]

1	newly 3 weeks spec	724.	469.935	{256., 720.}	44.2034	0.257287	newly 3 weeks cumul	3019.	1996.4	{1244., 2970.}	37.4173	0.215152	newly 5 weeks spec	306.	226.806	{123., 352.}	47.1432	0.245378	newly 5 weeks cumul	3848.	2558.98	{1550., 3882.}	39.5886	0.226568	newly 58 weeks cumul	4437.	3022.68	{1803., 4582.}	40.9111	0.229952
2	newly 3 weeks spec	551.	640.836	{424., 920.}	27.1756	0.103974	newly 3 weeks cumul	1831.	2195.57	{1605., 2818.}	26.0137	0.100398	newly 5 weeks spec	397.	459.836	{280., 684.}	36.078	0.135974	newly 5 weeks cumul	2702.	3196.33	{2201., 4273.}	25.7089	0.0973558	newly 54 weeks cumul	4210.	4882.03	{3292., 7006.}	30.5276	0.114959
3	newly 3 weeks spec	586.	551.049	{312., 910.}	36.7146	0.166065	newly 3 weeks cumul	2929.	2627.48	{1671., 4100.}	27.8711	0.134096	newly 5 weeks spec	256.	199.525	{106., 367.}	43.5899	0.228916	newly 5 weeks cumul	3549.	3162.15	{1970., 5082.}	30.0348	0.1444	newly 60 weeks cumul	3885.	3441.95	{2087., 5546.}	31.1142	0.149923
4	newly 3 weeks spec	581.	656.361	{416., 879.}	25.9445	0.10167	newly 3 weeks cumul	2166.	2267.52	{1643., 2801.}	16.6773	0.0711732	newly 5 weeks spec	320.	449.115	{248., 854.}	50.6865	0.155409	newly 5 weeks cumul	2954.	3273.7	{2173., 4232.}	22.4569	0.0906329	newly 54 weeks cumul	3942.	4779.31	{2893., 7935.}	33.0273	0.118579
5	newly 3 weeks spec	669.	312.516	{162., 569.}	58.3828	0.406916	newly 3 weeks cumul	2580.	1363.06	{726., 2448.}	52.3456	0.342218	newly 5 weeks spec	386.	149.887	{71., 277.}	64.7543	0.492285	newly 5 weeks cumul	3457.	1729.94	{929., 3128.}	54.9526	0.368795	newly 57 weeks cumul	4587.	2047.89	{1059., 3733.}	58.9671	0.417241
6	newly 3 weeks spec	808.	648.323	{396., 989.}	31.3957	0.163135	newly 3 weeks cumul	2954.	2647.18	{1762., 3750.}	20.9159	0.102208	newly 5 weeks spec	362.	335.5	{197., 503.}	38.4914	0.169111	newly 5 weeks cumul	3891.	3452.63	{2209., 4937.}	23.5452	0.115129	newly 58 weeks cumul	4944.	4143.29	{2598., 6143.}	28.6343	0.14374
7	newly 3 weeks spec	755.	555.167	{288., 973.}	43.6026	0.237251	newly 3 weeks cumul	2728.	2358.52	{1400., 3702.}	28.2545	0.140186	newly 5 weeks spec	406.	247.833	{110., 452.}	54.1215	0.333701	newly 5 weeks cumul	3682.	2978.92	{1691., 4832.}	34.0037	0.175097	newly 59 weeks cumul	4694.	3418.6	{1868., 5672.}	39.7344	0.2181
8	newly 3 weeks spec	735.	456.806	{239., 755.}	45.1788	0.279545	newly 3 weeks cumul	2632.	1801.16	{1014., 2772.}	37.0992	0.217839	newly 5 weeks spec	475.	247.887	{123., 437.}	53.416	0.358895	newly 5 weeks cumul	3719.	2389.31	{1300., 3782.}	41.1683	0.249305	newly 56 weeks cumul	5155.	3001.29	{1542., 4803.}	46.4898	0.294904
9	newly 3 weeks spec	675.	525.721	{323., 900.}	38.3534	0.200079	newly 3 weeks cumul	2453.	2027.92	{1357., 3209.}	28.6661	0.145332	newly 5 weeks spec	427.	286.77	{157., 517.}	47.4988	0.273195	newly 5 weeks cumul	3435.	2703.95	{1747., 4411.}	33.392	0.173886	newly 56 weeks cumul	4640.	3411.15	{2085., 5678.}	38.5938	0.208887
10	newly 3 weeks spec	697.	456.806	{239., 755.}	43.597	0.26244	newly 3 weeks cumul	2508.	1801.16	{1014., 2772.}	35.4864	0.203231	newly 5 weeks spec	467.	247.887	{123., 437.}	52.839	0.35247	newly 5 weeks cumul	3582.	2389.31	{1300., 3782.}	39.9313	0.237327	newly 56 weeks cumul	5280.	3001.29	{1542., 4803.}	47.3778	0.303643
11	newly 3 weeks spec	649.	535.767	{295., 775.}	26.1736	0.153266	newly 3 weeks cumul	2299.	1793.82	{1115., 2311.}	23.3239	0.143799	newly 5 weeks spec	447.	393.767	{192., 666.}	36.1596	0.18413	newly 5 weeks cumul	3330.	2649.48	{1642., 3715.}	24.7052	0.144691	newly 51 weeks cumul	4840.	4332.87	{2322., 6613.}	30.6329	0.151739
12	newly 3 weeks spec	799.	799.869	{558., 1086.}	19.5059	0.0878106	newly 3 weeks cumul	2755.	2689.26	{2100., 3213.}	14.281	0.0682638	newly 5 weeks spec	494.	539.475	{310., 895.}	31.9904	0.126247	newly 5 weeks cumul	3861.	3897.75	{2799., 4883.}	15.9544	0.0712296	newly 56 weeks cumul	5044.	5576.49	{3701., 7949.}	24.5941	0.0985234
13	newly 3 weeks spec	580.	583.767	{394., 787.}	22.7816	0.103481	newly 3 weeks cumul	2137.	2013.63	{1513., 2568.}	17.615	0.0902584	newly 5 weeks spec	319.	417.8	{246., 593.}	42.8422	0.143895	newly 5 weeks cumul	2908.	2936.48	{2098., 3803.}	20.6012	0.0929068	newly 52 weeks cumul	3866.	4628.58	{2929., 7007.}	33.1449	0.120825
14	newly 3 weeks spec	614.	447.242	{235., 819.}	44.7541	0.245457	newly 3 weeks cumul	2380.	1852.74	{1050., 3173.}	35.8173	0.188991	newly 5 weeks spec	361.	216.984	{107., 403.}	53.9139	0.330863	newly 5 weeks cumul	3257.	2382.24	{1355., 4145.}	39.971	0.218602	newly 57 weeks cumul	4210.	2850.08	{1576., 5027.}	44.1142	0.252406
15	newly 3 weeks spec	533.	562.082	{308., 907.}	32.3009	0.136096	newly 3 weeks cumul	2029.	1986.15	{1260., 2806.}	23.9931	0.108917	newly 5 weeks spec	261.	376.41	{180., 656.}	59.1169	0.177313	newly 5 weeks cumul	2645.	2826.11	{1676., 4442.}	29.5665	0.122627	newly 54 weeks cumul	3346.	4070.1	{2214., 6720.}	40.4359	0.144334
16	newly 3 weeks spec	754.	767.532	{505., 1086.}	23.9861	0.103049	newly 3 weeks cumul	2774.	2750.4	{2051., 3453.}	16.1692	0.0740855	newly 5 weeks spec	425.	477.871	{269., 825.}	38.1784	0.142147	newly 5 weeks cumul	3788.	3848.9	{2762., 5086.}	18.9444	0.0823466	newly 57 weeks cumul	4918.	5140.29	{3424., 7686.}	25.0233	0.103639
17	newly 3 weeks spec	715.	750.113	{439., 1231.}	29.427	0.120089	newly 3 weeks cumul	2771.	3026.06	{2047., 4254.}	21.1453	0.0855166	newly 5 weeks spec	457.	354.21	{193., 592.}	42.7155	0.22276	newly 5 weeks cumul	3767.	3909.4	{2486., 5831.}	22.6607	0.0951914	newly 59 weeks cumul	4791.	4585.27	{2841., 6973.}	24.8298	0.111803
18	newly 3 weeks spec	721.	730.323	{443., 1042.}	27.0145	0.116284	newly 3 weeks cumul	2736.	2688.77	{1898., 3475.}	17.1701	0.0778231	newly 5 weeks spec	402.	416.968	{216., 582.}	39.7609	0.161354	newly 5 weeks cumul	3695.	3660.97	{2419., 4889.}	21.8683	0.0973588	newly 57 weeks cumul	4724.	4666.27	{2857., 6304.}	25.9707	0.113958
19	newly 3 weeks spec	716.	877.242	{587., 1294.}	30.5123	0.111184	newly 3 weeks cumul	3184.	3307.77	{2497., 4077.}	16.1088	0.0722607	newly 5 weeks spec	379.	469.935	{256., 720.}	39.4842	0.133402	newly 5 weeks cumul	4076.	4426.94	{3132., 5549.}	19.0573	0.0776383	newly 59 weeks cumul	4764.	5453.21	{3734., 7571.}	24.0436	0.0918668
20	newly 3 weeks spec	765.	749.952	{447., 1127.}	26.4579	0.120707	newly 3 weeks cumul	2647.	2649.34	{1882., 3409.}	17.9662	0.0801999	newly 5 weeks spec	468.	456.806	{239., 755.}	38.5167	0.171708	newly 5 weeks cumul	3727.	3700.55	{2449., 5054.}	21.9099	0.0987096	newly 56 weeks cumul	5189.	4900.68	{2977., 7108.}	26.6159	0.124103
21	newly 3 weeks spec	673.	540.918	{334., 816.}	29.3352	0.15629	newly 3 weeks cumul	2256.	1921.9	{1279., 2669.}	22.6384	0.115108	newly 5 weeks spec	405.	367.459	{217., 547.}	36.357	0.172403	newly 5 weeks cumul	3166.	2749.2	{1934., 3980.}	24.6663	0.123588	newly 53 weeks cumul	4383.	4067.44	{2600., 6063.}	30.6733	0.142864
22	newly 3 weeks spec	723.	703.016	{392., 1186.}	35.1246	0.152664	newly 3 weeks cumul	3471.	3062.34	{2100., 4416.}	22.7052	0.111895	newly 5 weeks spec	278.	284.869	{140., 474.}	44.9286	0.180063	newly 5 weeks cumul	4204.	3806.3	{2491., 5918.}	26.224	0.124745	newly 60 weeks cumul	4711.	4234.48	{2683., 6601.}	28.8262	0.136719
23	newly 3 weeks spec	587.	542.905	{318., 911.}	30.5184	0.143056	newly 3 weeks cumul	1900.	2000.13	{1346., 2943.}	24.3208	0.101502	newly 5 weeks spec	330.	338.	{173., 594.}	41.3179	0.169452	newly 5 weeks cumul	2657.	2767.81	{1776., 4286.}	27.0522	0.113477	newly 55 weeks cumul	3467.	3787.41	{2239., 6271.}	33.1754	0.129411
24	newly 3 weeks spec	579.	481.705	{276., 812.}	34.6697	0.176314	newly 3 weeks cumul	2054.	1759.36	{1127., 2719.}	26.5559	0.13361	newly 5 weeks spec	382.	310.328	{161., 597.}	44.906	0.229158	newly 5 weeks cumul	2904.	2460.41	{1481., 4072.}	30.5813	0.154098	newly 53 weeks cumul	4101.	3465.67	{1988., 6335.}	36.2331	0.179711
25	newly 3 weeks spec	727.	799.869	{558., 1086.}	22.7907	0.0934975	newly 3 weeks cumul	3100.	2689.26	{2100., 3213.}	15.275	0.0798094	newly 5 weeks spec	309.	539.475	{310., 895.}	76.6884	0.219254	newly 5 weeks cumul	3847.	3897.75	{2799., 4883.}	16.0602	0.0714365	newly 56 weeks cumul	4343.	5576.49	{3701., 7949.}	35.0589	0.124976
26	newly 3 weeks spec	523.	649.94	{339., 1141.}	43.1181	0.153723	newly 3 weeks cumul	2145.	2721.21	{1614., 4006.}	38.5958	0.139517	newly 5 weeks spec	188.	327.985	{133., 538.}	86.2417	0.213429	newly 5 weeks cumul	2653.	3520.7	{1992., 5557.}	43.5778	0.150573	newly 59 weeks cumul	2904.	4156.1	{2231., 6616.}	53.1531	0.169841
27	newly 3 weeks spec	757.	767.532	{505., 1086.}	23.9549	0.103327	newly 3 weeks cumul	2773.	2750.4	{2051., 3453.}	16.1738	0.0740804	newly 5 weeks spec	463.	477.871	{269., 825.}	37.1212	0.151548	newly 5 weeks cumul	3879.	3848.9	{2762., 5086.}	18.6014	0.0827903	newly 57 weeks cumul	5146.	5140.29	{3424., 7686.}	24.7765	0.107459
28	newly 3 weeks spec	747.	392.912	{184., 729.}	53.5515	0.358922	newly 3 weeks cumul	3109.	1966.57	{1007., 3396.}	43.332	0.264627	newly 5 weeks spec	347.	141.515	{67., 244.}	63.1844	0.473728	newly 5 weeks cumul	3954.	2349.93	{1174., 4056.}	46.6419	0.293112	newly 60 weeks cumul	4582.	2554.1	{1254., 4404.}	49.3636	0.319656
29	newly 3 weeks spec	724.	762.984	{568., 962.}	17.8924	0.0756914	newly 3 weeks cumul	2790.	2519.82	{1830., 3037.}	14.3751	0.0731024	newly 5 weeks spec	343.	557.066	{317., 957.}	66.0613	0.196693	newly 5 weeks cumul	3611.	3733.25	{2772., 4555.}	14.706	0.0635063	newly 55 weeks cumul	4378.	5687.97	{3753., 8561.}	35.4477	0.124501
30	newly 3 weeks spec	685.	656.361	{416., 879.}	23.1112	0.106489	newly 3 weeks cumul	2400.	2267.52	{1643., 2801.}	15.2766	0.0720099	newly 5 weeks spec	366.	449.115	{248., 854.}	41.2165	0.140723	newly 5 weeks cumul	3210.	3273.7	{2173., 4232.}	19.5526	0.085498	newly 55 weeks cumul	4078.	4779.31	{2893., 7935.}	30.8605	0.113872
31	newly 3 weeks spec	731.	649.226	{408., 989.}	28.057	0.133994	newly 3 weeks cumul	2627.	2648.35	{1800., 3750.}	20.0474	0.0872053	newly 5 weeks spec	439.	335.774	{197., 503.}	43.2287	0.221714	newly 5 weeks cumul	3631.	3454.06	{2243., 4937.}	22.1604	0.101181	newly 58 weeks cumul	4700.	4144.56	{2615., 6143.}	26.6771	0.127891
32	newly 3 weeks spec	583.	543.867	{310., 775.}	25.0314	0.133754	newly 3 weeks cumul	2074.	1817.13	{1179., 2344.}	19.6191	0.116089	newly 5 weeks spec	399.	398.367	{197., 666.}	36.8505	0.167515	newly 5 weeks cumul	2998.	2684.43	{1649., 3715.}	22.4661	0.122052	newly 50 weeks cumul	4177.	4389.63	{2417., 6613.}	31.8738	0.134983
33	newly 3 weeks spec	525.	649.226	{408., 989.}	34.6237	0.121213	newly 3 weeks cumul	2485.	2648.35	{1800., 3750.}	21.0917	0.0867979	newly 5 weeks spec	210.	335.774	{197., 503.}	64.2089	0.173578	newly 5 weeks cumul	3022.	3454.06	{2243., 4937.}	26.053	0.0995466	newly 58 weeks cumul	3330.	4144.56	{2615., 6143.}	33.2776	0.115791
34	newly 3 weeks spec	536.	335.148	{182., 606.}	48.8378	0.294314	newly 3 weeks cumul	2500.	1732.21	{1018., 2896.}	41.7043	0.236956	newly 5 weeks spec	238.	119.197	{60., 207.}	56.7227	0.385841	newly 5 weeks cumul	3124.	2050.93	{1184., 3484.}	44.1652	0.258776	newly 60 weeks cumul	3558.	2211.54	{1251., 3736.}	46.273	0.27942
35	newly 3 weeks spec	625.	422.295	{219., 744.}	44.4354	0.258065	newly 3 weeks cumul	2116.	1630.79	{948., 2619.}	34.3658	0.185119	newly 5 weeks spec	382.	249.443	{118., 458.}	51.678	0.305025	newly 5 weeks cumul	2967.	2206.3	{1211., 3639.}	38.4387	0.210616	newly 55 weeks cumul	4257.	2887.23	{1525., 4858.}	44.238	0.25469
36	newly 3 weeks spec	695.	541.617	{342., 762.}	28.9952	0.155247	newly 3 weeks cumul	2481.	1920.67	{1311., 2606.}	24.9926	0.133608	newly 5 weeks spec	436.	367.133	{205., 565.}	36.445	0.180181	newly 5 weeks cumul	3487.	2738.1	{1847., 3877.}	26.5156	0.140616	newly 53 weeks cumul	5127.	4054.7	{2496., 5746.}	32.1884	0.168085
37	newly 3 weeks spec	692.	671.	{445., 1107.}	26.9852	0.119222	newly 3 weeks cumul	2700.	2473.75	{1801., 3149.}	17.5756	0.0847012	newly 5 weeks spec	388.	387.951	{210., 721.}	37.219	0.155566	newly 5 weeks cumul	3670.	3385.52	{2327., 5130.}	22.0418	0.103923	newly 56 weeks cumul	4686.	4377.21	{2851., 6909.}	26.3932	0.120645
38	newly 3 weeks spec	526.	750.113	{439., 1231.}	49.2733	0.157123	newly 3 weeks cumul	2700.	3026.06	{2047., 4254.}	22.454	0.0887458	newly 5 weeks spec	147.	354.21	{193., 592.}	140.959	0.333256	newly 5 weeks cumul	3166.	3909.4	{2486., 5831.}	31.257	0.112868	newly 59 weeks cumul	3369.	4585.27	{2841., 6973.}	41.6956	0.139456
39	newly 3 weeks spec	662.	635.25	{382., 948.}	34.076	0.148928	newly 3 weeks cumul	2825.	2726.03	{1835., 3866.}	24.7681	0.111633	newly 5 weeks spec	284.	277.117	{160., 419.}	39.689	0.17073	newly 5 weeks cumul	3546.	3426.08	{2234., 4868.}	27.5602	0.122824	newly 59 weeks cumul	4001.	3896.87	{2412., 5536.}	29.4193	0.129399
40	newly 3 weeks spec	671.	674.279	{448., 1107.}	26.2833	0.111901	newly 3 weeks cumul	2384.	2482.2	{1801., 3149.}	17.931	0.0766549	newly 5 weeks spec	421.	389.262	{210., 721.}	37.6854	0.170026	newly 5 weeks cumul	3361.	3397.18	{2327., 5130.}	21.133	0.0914383	newly 56 weeks cumul	4464.	4391.15	{2851., 6909.}	25.2828	0.10991
41	newly 3 weeks spec	690.	539.475	{310., 895.}	35.2768	0.187143	newly 3 weeks cumul	2492.	2008.36	{1315., 2923.}	26.9281	0.14223	newly 5 weeks spec	409.	326.066	{154., 562.}	43.5488	0.225713	newly 5 weeks cumul	3447.	2756.72	{1699., 4339.}	30.8831	0.163031	newly 55 weeks cumul	4485.	3687.1	{2125., 6016.}	34.6978	0.177199
42	newly 3 weeks spec	566.	517.667	{318., 816.}	29.6623	0.141736	newly 3 weeks cumul	1986.	1787.62	{1210., 2444.}	22.0864	0.107735	newly 5 weeks spec	394.	364.286	{182., 652.}	40.7703	0.190787	newly 5 weeks cumul	2865.	2593.57	{1597., 3955.}	26.4484	0.128149	newly 50 weeks cumul	4248.	3955.52	{2248., 6358.}	33.1009	0.154087
43	newly 3 weeks spec	779.	703.016	{392., 1186.}	36.8	0.17155	newly 3 weeks cumul	3579.	3062.34	{2100., 4416.}	23.8953	0.120162	newly 5 weeks spec	296.	284.869	{140., 474.}	46.0844	0.197483	newly 5 weeks cumul	4369.	3806.3	{2491., 5918.}	27.5874	0.135162	newly 60 weeks cumul	4830.	4234.48	{2683., 6601.}	29.6748	0.143574
44	newly 3 weeks spec	659.	599.413	{399., 843.}	23.152	0.112464	newly 3 weeks cumul	2196.	1997.49	{1465., 2539.}	16.8922	0.0817286	newly 5 weeks spec	414.	441.667	{249., 763.}	36.5578	0.15007	newly 5 weeks cumul	3184.	2956.83	{2029., 3957.}	20.1184	0.0955727	newly 51 weeks cumul	4755.	4683.06	{2789., 7168.}	29.0201	0.128575
45	newly 3 weeks spec	602.	669.016	{420., 1007.}	27.8362	0.111765	newly 3 weeks cumul	2275.	2378.52	{1645., 3125.}	19.3399	0.0832575	newly 5 weeks spec	418.	422.295	{219., 744.}	36.6774	0.155022	newly 5 weeks cumul	3189.	3340.3	{2242., 4701.}	21.8368	0.093216	newly 55 weeks cumul	4496.	4596.74	{2819., 7039.}	26.6295	0.113433
46	newly 3 weeks spec	631.	233.017	{106., 364.}	65.9192	0.510067	newly 3 weeks cumul	2297.	895.4	{453., 1329.}	62.6542	0.468741	newly 5 weeks spec	464.	143.083	{71., 188.}	71.1818	0.592175	newly 5 weeks cumul	3301.	1222.38	{610., 1749.}	64.7203	0.495369	newly 53 weeks cumul	4939.	1681.17	{802., 2356.}	67.7755	0.539222
47	newly 3 weeks spec	796.	762.984	{568., 962.}	16.2905	0.0756131	newly 3 weeks cumul	2859.	2519.82	{1830., 3037.}	14.8235	0.076597	newly 5 weeks spec	468.	557.066	{317., 957.}	35.3125	0.12845	newly 5 weeks cumul	3902.	3733.25	{2772., 4555.}	13.5878	0.0633203	newly 55 weeks cumul	5202.	5687.97	{3753., 8561.}	23.4866	0.0939929
48	newly 3 weeks spec	746.	792.115	{463., 1225.}	29.5324	0.122815	newly 3 weeks cumul	2942.	2945.03	{2065., 3837.}	17.855	0.0795704	newly 5 weeks spec	435.	434.033	{214., 804.}	42.7473	0.184069	newly 5 weeks cumul	3949.	3980.64	{2603., 5680.}	22.3489	0.098401	newly 58 weeks cumul	4973.	4921.52	{3021., 7679.}	26.8756	0.118606
49	newly 3 weeks spec	753.	777.183	{432., 1264.}	30.29	0.126917	newly 3 weeks cumul	3170.	3058.48	{2010., 4064.}	17.7419	0.0823605	newly 5 weeks spec	359.	372.567	{183., 678.}	42.9712	0.1726	newly 5 weeks cumul	4008.	3986.22	{2505., 5933.}	22.4206	0.0996959	newly 59 weeks cumul	4662.	4673.73	{2783., 7225.}	25.7164	0.111628
50	newly 3 weeks spec	849.	827.918	{577., 1086.}	18.1274	0.0828284	newly 3 weeks cumul	3183.	2898.97	{2262., 3389.}	12.4318	0.0606963	newly 5 weeks spec	465.	525.721	{323., 900.}	34.0208	0.126354	newly 5 weeks cumul	4282.	4098.97	{3110., 5053.}	14.0301	0.065519	newly 57 weeks cumul	5322.	5482.2	{3843., 7962.}	20.3609	0.0859938
